# Zinc in the Monoaminergic Theory of Depression: Its Relationship to Neural Plasticity

**DOI:** 10.1155/2017/3682752

**Published:** 2017-02-19

**Authors:** Urszula Doboszewska, Piotr Wlaź, Gabriel Nowak, Maria Radziwoń-Zaleska, Ranji Cui, Katarzyna Młyniec

**Affiliations:** ^1^Department of Pharmacobiology, Jagiellonian University Medical College, Medyczna 9, 30-688 Kraków, Poland; ^2^Department of Animal Physiology, Institute of Biology and Biochemistry, Faculty of Biology and Biotechnology, Maria Curie-Sklodowska University, Akademicka 19, 20-033 Lublin, Poland; ^3^Institute of Pharmacology, Polish Academy of Sciences, Smętna 12, 31-343 Kraków, Poland; ^4^Department of Psychiatry, Medical University of Warsaw, Nowowiejska 27, 00-665 Warszawa, Poland; ^5^Jilin Provincial Key Laboratory on Molecular and Chemical Genetics, The Second Hospital of Jilin University, Changchun, Jilin 130041, China

## Abstract

Preclinical and clinical studies have demonstrated that zinc possesses antidepressant properties and that it may augment the therapy with conventional, that is, monoamine-based, antidepressants. In this review we aim to discuss the role of zinc in the pathophysiology and treatment of depression with regard to the monoamine hypothesis of the disease. Particular attention will be paid to the recently described zinc-sensing GPR39 receptor as well as aspects of zinc deficiency. Furthermore, an attempt will be made to give a possible explanation of the mechanisms by which zinc interacts with the monoamine system in the context of depression and neural plasticity.

## 1. Introduction

The original monoamine hypothesis of depression was based on serendipitous discoveries. Clinical observations on iproniazid, a tuberculostatic, and imipramine, which was designed as a neuroleptic, showed that these medications reduce depressive symptoms [1]. Extensive research on their mechanism of action further revealed that iproniazid inhibits monoamine oxidase (MAO), an enzyme responsible for the oxidative deamination of monoamines, such as norepinephrine (NE) and serotonin (5-hydroxytryptamine, 5-HT), while imipramine, which became the first tricyclic antidepressant (TCA), inhibits the serotonin transporter (SERT) and the norepinephrine transporter (NET), which account for clearance of the neurotransmitters from the synaptic cleft [1]. These and other observations have contributed to the monoamine hypothesis, which postulated that depression is associated with decreased levels of NE and/or 5-HT in the brain [2, 3]. Although the monoamine hypothesis is now regarded as too simplistic to explain the complexity of the pathophysiology of depression, it has led to the development of antidepressants such as selective serotonin reuptake inhibitors (SSRIs) or serotonin-norepinephrine reuptake inhibitors (SNRIs), which are now widely used. It should be noted that almost all currently used antidepressant drugs target the monoamine system. However, the Sequenced Treatment Alternatives to Relieve Depression (STAR^*∗*^D) study, the largest and longest study conducted with the aim of determining antidepressants effectiveness, demonstrated that only one-third of the participants given an SSRI as a first-line treatment reached remission, and that about 10–15% more responded [4]. These data emphasize the need for novel pharmacological treatments and/or augmentation strategies for depression. It has been shown in preclinical and clinical studies that zinc possesses antidepressant properties [5] and is able to enhance the effects of antidepressant drugs belonging to the group of SSRIs or TCAs [6, 7]. This review aims to discuss the role of zinc in the pathophysiology and treatment of the disease with regard to the monoamine hypothesis. Particular attention will be paid to the recently described zinc-sensing GPR39 receptor [8] and aspects of zinc deficiency. Furthermore, an attempt will be made to give a possible explanation of the mechanisms by which zinc interacts with the monoamine system in the context of depression and neural plasticity.

## 2. Zinc

Recent years have brought a new evidence supporting the involvement of zinc in depression in terms of both pharmacological and clinical/epidemiological data. Preclinical tests and models of depression showed antidepressant-like activity of zinc [9–15]. Clinical data pointed towards potential benefits of zinc administration in depressed patients [16, 17]. Zinc supplementation was shown to be effective as an adjunct therapy [6, 7, 18] or as a stand-alone intervention [19, 20] for depression. Moreover, the intake of zinc was suggested to be among the dietary factors that may be associated with a risk for depression. Studies performed in rodents indicated a causative role of dietary zinc restriction in the induction of depressive-like symptoms [21–24] or anhedonia [25–27]. Some large, cross-sectional, population-based epidemiological studies have suggested that a low dietary zinc intake is associated with depression in women [28, 29], but not in men [28]. Although the first prospective study aimed to examine the association of zinc intake and depression risk demonstrated a modest, but significant, inverse correlation between the intake of this element and depression in a cross-sectional setting, the 20-year prospective follow-up observations have indicated that a low dietary zinc intake may not precede depression in initially depression-free men [30]. However, because the study sample comprised exclusively men who have received a hospital discharge diagnosis of unipolar depression, the results cannot be generalized to women or patients not warranting hospitalization [30]. In contrast, in a prospective study of both men and women, a low dietary zinc intake emerged as a risk factor for depression [31]. Also mice lacking the GPR39 receptor, a G protein-coupled receptor, which is activated by zinc [8, 32], display depressive-like behavior [33]. Recently, TC G-1008, an agonist of GPR39, was found to exhibit antidepressant-like activity [34]. These observations further support the involvement of zinc in the treatment of depression.

In the human body zinc is the second most prevalent trace element. It is necessary for proper brain development and functioning. Even subclinical zinc deficiency impairs human brain function [35]. In the brain the ion is present in presynaptic vesicles of a subset of glutamatergic neurons [36]. Somas of the zinc containing neurons are located in the cerebral cortex and in the amygdala, whereas their axonal projections reach cerebral cortex and amygdala, striatum as well as structures of the limbic system [37]. Zinc is packed into the synaptic vesicles by means of a zinc transporter 3 (ZnT-3) [38], which is localized on the membranes of the vesicles [39]. After action potential it is released from the presynaptic vesicles into the synaptic cleft. To date, there is no consensus on the concentration to which the ion rises in the synaptic cleft or its time-course. It is estimated to range from sub-*μ*M to over 100 *μ*M [40]. Zinc released from the presynaptic vesicles modulates a variety of receptors or transporters on the postsynaptic side, including those for monoamines [41, 42].

## 3. Serotonin (5-HT)

In 1967 Coppen emphasized the role of 5-HT in the pathophysiology of depression [2]. Approximately 90% of 5-HT is synthesized by enterochromaffin cells in the gastrointestinal tract, while most of the remaining 5-HT is produced by the neurons of the raphe nuclei in the brain. The axonal projections of the raphe nuclei innervate almost the entire central nervous system (CNS), and thus play an important role in the regulation of mood, memory, cognition, sleep, appetite, and so forth. 5-HT is synthesized from the precursor amino acid L-tryptophan [43, 44]. After tryptophan is transported into the 5-HT neuron, it is converted by tryptophan hydroxylase, the rate-limiting enzyme in 5-HT production. Aromatic amino acid decarboxylase then converts 5-hydroxytryptophan into 5-HT, which is taken up into the synaptic vesicles by the vesicular monoamine transporter (VMAT2) [45]. Tryptophan can be also catabolized by indoleamine 2,3-dioxygenase (IDO) into kynurenine, which is further broken down into kynurenic acid and quinolinic acid [46]. Activation of the IDO pathway causes lowered plasma tryptophan availability to the brain and, subsequently, lower production of 5-HT [47]. MAO, which is localized in the mitochondrial outer membrane, degrades 5-HT [46]. SERT, which belongs to the neurotransmitter/sodium symporter family besides NET and dopamine transporter (DAT), is a high-affinity transporter for 5-HT and plays a crucial role in maintaining its extracellular levels [48]. Drugs that selectively inhibit SERT, namely, SSRIs, have revolutionized clinical psychopharmacology and nowadays are among the first-line medications used for depression [4]. Although the newest drugs, such as the multimodal agent vortioxetine, combine action occurring at different 5-HT receptor subtypes, SERT inhibition remains an important element of their action [49]. The current view on the mechanism of action of SSRIs states that, following acute treatment, the 5-HT level rises in the somatodendritic area located in the raphe nuclei and stimulates the 5-HT_1A_ autoreceptors. Following chronic use, the 5-HT_1A_ receptors become downregulated and desensitized and thus no longer inhibit 5-HT release. This leads to increased 5-HT release from the axon terminals [50]. As well as targeting SERT, vortioxetine targets 5-HT G-protein-coupled (5-HT_1A_ and 5-HT_1B_ partial agonism and 5-HT_7_ antagonism) and 5-HT ionotropic (5-HT_3_ antagonism) receptors [49]. Other medications, such as trazodone, as well as inhibiting SERT, possess 5-HT_2A_ and 5-HT_2C_ antagonistic properties [51].

### 3.1. The Interaction of Zinc with the Serotonergic System

Several preclinical studies have shown the interaction of zinc with the components of the serotonergic system with regard to its antidepressant-like action. Pretreatment with p-chlorophenylalanine (pCPA), an inhibitor of 5-HT synthesis, abolished the antidepressant-like effect of zinc in the forced swim test (FST), which is a common preclinical paradigm used to assess antidepressant properties [52]. The antidepressant-like effect of zinc in the FST was also blocked by the 5-HT_1A_ receptor antagonist WAY-100635 [52]. A study by Cichy et al. [53] showed adaptive changes in serotonergic receptors following chronic zinc hydroaspartate administration. Treatment with zinc for 2 weeks increased the density of the hippocampal and cortical 5-HT_1A_ and 5-HT_2A_ receptors in rats. Similarly, adaptive changes of 5-HT_1A_ and 5-HT_2A_ receptors were found following chronic administration of imipramine [54].

Effect on serotonergic receptors is one of the explanations of the antidepressant-like properties of zinc, which are observed in both preclinical and clinical studies. Satała et al. [42] extensively explored the pharmacological profile of zinc at the 5-HT_1A_ receptors using the agonist of 5-HT_1A_, [3H]-8-OH-DPAT. In this study, the effects of zinc on [3H]-8-OH-DPAT binding to 5-HT_1A_ stably expressed in HEK293 cells were investigated by means of the in vitro radioligand binding method. A biphasic effect, which involved allosteric potentiation of agonist binding at sub-*μ*M zinc concentrations and inhibition at sub-mM zinc concentrations, was observed [42]. Given that it is estimated that after depolarization the concentration of zinc in the synaptic cleft ranges from sub-*μ*M to 100 *μ*M [40], the effects observed with lower doses should be physiologically more relevant. Additionally, in vivo studies, aimed at differentiating between action at pre- and postsynaptic 5-HT_1A_, demonstrated that zinc did not induce lower lip retraction or elements of behavioral syndrome (flat body posture, forepaw treading), but pretreatment with zinc blocked these effects induced by the agonist [3H]-8-OH-DPAT, which suggest that zinc may act as an antagonist of this receptor at the postsynaptic site. However, zinc decreased body temperature similarly to [3H]-8-OH-DPAT. On the other hand, the experiments using 5-HT_1A_ autoreceptor knockout mice showed that lack of this receptor completely blocked the hypothermia induced by zinc, while in wild-type littermate mice a consistent decrease in body temperature was observed, which may indicate agonist-like profile at the presynaptic 5-HT_1A_. In the FST ineffective doses of zinc potentiated the effects of ineffective doses of [3H]-8-OH-DPAT. In the FST conducted with 5-HT_1A_ autoreceptor knockout mice zinc induced a slight decrease in the immobility time while in wild-type littermates a significant decrease in the immobility time was observed, which suggest that presynaptic 5-HT_1A_ receptors are necessary for the antidepressant-like effect of zinc [42]. To sum up, this comprehensive study shows that zinc (depending on its concentration) may act as positive allosteric modulator of agonist binding to 5-HT_1A_ receptors or inhibitor. Moreover, both agonist and antagonist-like effects were found and it may target both pre- and postsynaptic 5-HT_1A_.

A 10-day administration of pCPA, an inhibitor of 5-HT synthesis, caused downregulation of GPR39 protein in the hippocampus of mice [55], which suggests a link between GPR39 and 5-HT signaling. There was also a decrease in the level of 5-HT precursor, tryptophan, in the hippocampus of GPR39 knockout mice [55]. Moreover, there is some evidence indicating a link between GPR39 and 5-HT_1A_ receptors function. GPR39 was found to form heterodimers with 5-HT_1A_ as well as heterotrimers with 5-HT_1A_ and galanin receptor 1 (GalR_1_) upon coexpression of the two or three of them in mammalian cells [56]. Galanin is a neuropeptide which is widely distributed in the brain and whose effects are mediated via three G protein-coupled receptors: GalR_1_, GalR_2_, and GalR_3_ [57]. 5-HT_1A_ and GalR_1_, which share the same signal transduction pathway (i.e., activate G_*α*i_ protein which leads to inhibition of adenylyl cyclase), have been described to heterodimerize and functional characteristic of the heterodimers revealed absence of addictive effects what can be explained by the existence of allosteric antagonistic communication to avoid excessive inhibition of adenylyl cyclase [58]. Furthermore, zinc was found to disrupt the heterodimerization process of 5-HT_1A_ and GalR_1_ [59]. Activities of the monohomomeric receptors: 5-HT_1A_, GalR_1_, and GPR39, and the heteroreceptor complexes: 5-HT_1A_-GPR39 and 5-HT_1A_-GPR39-GalR_1_, were measured by their ability to activate response elements: the serum response element (SRE) and nuclear factor kappa beta response element (NF*κβ*-RE) [56]. Because GPR39 signals via G_q_, G_*α*12/13_, and G_*α*s_ proteins [32], which leads to activation of both response elements, whereas 5-HT_1A_ signals via G_*α*i_ protein [60] and activates SRE only in the presence of agonist, 8-OH-DPAT, SRE, and NF*κβ*-RE were chosen to analyze potential differences in signaling between monohomomers and heteromers [56]. 5-HT_1A_-GPR39 heteromer exposure to 8-OH-DPAT and zinc chloride resulted in higher response compared to those yielded by each one of the compounds indicating additive signaling upon coactivation of these receptors. The complex including also GalR_1_ (5-HT_1A_-GPR39-GalR_1_ heteromer) displayed no response in the presence of 8-OH-DPAT or zinc chloride, but enhancement of response was observed in the presence of galanin, while stimulation with all agonists together evoked the same response as galanin, suggesting that the presence of GalR_1_ blocks 5-HT_1A_ and GPR39 signaling.

In addition to targeting 5-HT_1A_ receptors, zinc was found to target other subtypes of 5-HT receptors. Electrophysiological studies of HEK293 cells expressing 5-HT_3_ receptors showed that low concentrations of zinc (0.3–10 *μ*M) enhanced and high concentrations of zinc (30–200 *μ*M) depressed the 5-HT-induced response [61]. 5-HT reuptake by SERT is not affected by zinc [62].

### 3.2. Interactions between Zinc and Antidepressants Targeting the Serotonergic System

Preclinical and clinical studies showed that zinc interacts with the serotonergic system and therefore enhances antidepressant-like effects. Joint administration of zinc with SSRIs such as fluoxetine or citalopram (all in subeffective doses) produced an antidepressant-like effect in the FST [52]. Moreover, an increase in the swimming parameter, but not in the climbing parameter, in the FST was observed following zinc administration [52]. It is suggested that in the FST the swimming parameter is connected with serotonergic neurotransmission, and the climbing is linked to noradrenergic neurotransmission (based on the observation that SSRIs increase the swimming time whereas selective norepinephrine reuptake inhibitors (NRIs) increase the climbing time [63]). Thus, these results suggest that the serotonergic system is involved in the antidepressant-like activity of zinc. Combined administration of zinc chloride with SSRIs such as fluoxetine or paroxetine significantly reduced immobility scores in the tail suspension test (TST), another preclinical paradigm used to assess antidepressant activity, without affecting locomotion in the open field test [13]. Moreover, joint administration of zinc and imipramine (both in ineffective doses) caused an antidepressant-like effect in the chronic unpredictable stress (CUS) protocol, which is a commonly used preclinical model of depression [14]. Imipramine was also found to be active in the FST following the administration of ineffective doses together with ineffective doses of zinc sulfate [11, 12]. Wróbel et al. [64] demonstrated antidepressant-like properties of zinc in a dexamethasone-induced model of depression in mice. In this study, the joint administration of zinc and imipramine (both in ineffective doses) reversed dexamethasone-induced depressive-like behavior, as measured by the FST.

The interaction between zinc and antidepressants targeting the serotonergic system has been observed not only following zinc supplementation, but also under zinc-deficient conditions. Chronic administration of the zinc-deficient diet was found to alter the responsiveness to antidepressant drugs [65]. Animals subjected to a zinc-deficient diet and treated with an acute injection of escitalopram or imipramine displayed increased immobility time in the FST, compared to animals treated with a zinc-adequate diet and the respective antidepressant agent [65]. Additionally, increased immobility time was observed in mice that received a zinc-deficient diet and chronic treatment with escitalopram, compared to mice that received a zinc-adequate diet and the drug, whereas chronic imipramine treatment did not result in such differences between the zinc-deficient and zinc-adequate rats [65]. Chronic dietary deprivation of zinc produces a depressive- and anxiety-like phenotype [22–24, 66, 67]. Chronic administration of fluoxetine to the zinc-deficient rats resulted in the normalization of depressive-like behavior induced by the diet, as measured by decreased immobility time in the FST [66]. On the contrary, Tassabehji et al. [25] did not observe a significant reduction in the immobility time in the zinc-deficient rats treated with fluoxetine, compared to zinc-deficient rats that did not receive the antidepressant. However, Tassabehji et al. [25] also did not observe depressive-like behavior (increased immobility time) in the zinc-deficient rats compared to control rats. This finding may have resulted from different study design. In the study of Doboszewska et al. [66] the rats received the zinc-deficient diet for 4 weeks (i.e., for a period of time after which behavioral (increased immobility time in the FST, anhedonia) and neurobiological changes associated with depression are established [27]) and for subsequent 2 weeks they received fluoxetine (10 mg/kg/day intraperitoneally (i.p.)) in addition to the diet, whereas in the study of Tassabehji et al. [25] the rats received the zinc-deficient diet and fluoxetine (10 mg/kg/day via drinking water) for 3 weeks. Therefore, a shorter duration of the zinc-deficient diet in the study of Tassabehji et al. [25] (3 weeks) may have been an insufficient amount of time in which to observe a depressive-like behavior. Yet other authors demonstrated depressive-like behavior in the FST following a shorter duration of the zinc-deficient diet, namely, 2 weeks [21, 68]. It should be noted that Tassabehji et al. [25] observed anhedonia, which is one of the core symptoms of depression, in the zinc-deficient rats. Whereas the two studies in which increased immobility time in the FST following 2 weeks of the diet utilized the zinc-deficient diet containing 0.37 mg zinc/kg and the zinc-adequate diet containing 52.8 mg zinc/kg [21, 68], similarly, we utilized the following diets: zinc-deficient 3 mg zinc/kg; zinc-adequate 50 mg zinc/kg [66], the study which did not demonstrate a depressive-like behavior after 3 weeks of the diet utilized: zinc-deficient diet 1 mg/kg; zinc-adequate diet 30 mg/kg [25]. Hence, when interpreting data on the effectiveness of antidepressants in the zinc-deficient animals it is important to take into account the duration of the diet, the amount of the ion, and the schedule of treatment. Positive behavioral effects (reversal of depressive-like behavior) in zinc-deficient mice were observed following chronic treatment with desipramine (a TCA with a less potent inhibitory effect on 5-HT than on NA reuptake) in the FST and the TST [22].

Administration of antidepressants that influence the serotonergic system under zinc-deficient conditions also normalized changes that were observed in the brain. Fluoxetine prevented the higher levels of the hippocampal N-methyl-D-aspartate receptor (NMDAR) subunits (GluN1, GluN2A, GluN2B) and the reduced levels of phosphorylated on Serine-845 GluA1 subunit of alpha-amino-3-hydroxy-5-methyl-4-isoxazole-propionic acid receptor (AMPAR) (pS485-GluA1), phosphorylated cyclic AMP response element binding protein (p-CREB) and brain-derived neurotrophic factor (BDNF) that were evoked by zinc deficiency [66]. Chronic desipramine treatment normalized the exaggerated immediate-early gene expression in the amygdala that was induced by zinc deficiency [22].

There is also a link between GPR39 receptor and antidepressants acting on the 5-HT system. Chronic (a 2-week) treatment with escitalopram but not with imipramine induced upregulation of GPR39 receptor at the protein level in the frontal cortex of mice [69]. Moreover, acute administration of imipramine or escitalopram to mice fed with the zinc-deficient diet caused downregulation of GPR39 protein, while chronic administration of these agents (which is required in order to relieve depressive symptoms) induced upregulation of this protein in the frontal cortex of mice [70]. Furthermore, imipramine and escitalopram reduced immobility time in the FST in wild-type mice, but they were inactive in this paradigm in GPR39 knockout mice, which suggest that GPR39 receptor is necessary for the antidepressant effect of drugs targeting the 5-HT system [71].

Besides preclinical studies, clinical studies have also demonstrated the interaction between zinc and antidepressants targeting the serotonergic system. The first clinical report indicating the beneficial effects of zinc in human depression showed that it is effective as an augmentation strategy in conjunction with TCAs (clomipramine, amitriptyline) or SSRIs (citalopram, fluoxetine) [6]. This preliminary observation was further confirmed in trials using a bigger sample size. The study of Ranjbar et al. showed that zinc supplementation of therapy involving administration of SSRIs, citalopram or fluoxetine, reduced major depressive disorder symptoms more effectively than administration of the respective drug plus placebo [7]. This effect was not associated with changes in plasma levels of IL-6, TNF-*α* or BDNF [72]. Importantly, zinc supplementation of therapy involving administration of imipramine was found to be more effective than administration of imipramine plus placebo in treatment-resistant patients [18]. Although a recent systematic review and meta-analysis of adjunctive nutraceuticals for depression found mixed results for zinc [17], zinc supplementation shows promise as a strategy for improving an inadequate response to antidepressants.

### 3.3. Effects on Zinc Levels of Antidepressants Targeting the Serotonergic System

In preclinical studies, chronic treatment with citalopram (but not with imipramine) significantly increased the serum zinc level. Chronic treatment with both drugs slightly increased the zinc level in the hippocampus and slightly decreased it in the cortex, the cerebellum and the basal forebrain [73]. Moreover, escitalopram and imipramine normalized serum zinc levels previously reduced by a 6-week zinc-deficient diet [65]. Also, chronic treatment with fluoxetine normalized a decrease in the serum zinc level induced by dietary zinc deficiency [66].

A clinical study by Maes et al. [74] examining the serum zinc level in treatment-resistant depression showed a decreased serum zinc level in treatment-resistant patients compared with healthy controls and patients who were not resistant to treatment. The study also showed that subsequent treatment with antidepressants for 5 weeks (with trazodone alone or in combination with fluoxetine and pindolol) did not induce significant changes in the level of serum zinc. Therefore, the serum zinc level was proposed as a marker for treatment resistance. Moreover, a study of the use of zinc supplementation in imipramine therapy showed significantly lower serum zinc level in depressed patients than in healthy volunteers. All groups demonstrated a gradual increase in zinc concentrations over the period of treatment with imipramine with or without zinc supplementation. It is of note that treatment-resistant patients demonstrated lower concentrations of zinc than patients who were not resistant to treatment. Importantly, following 12 weeks of treatment with imipramine, a significant negative correlation was demonstrated between the Montgomery-Åsberg Depression Rating Scale and the serum zinc level, together with a concomitant increase in serum zinc in patients in remission, which suggests that the serum zinc level is a state marker for depression (with the exception of treatment-resistant patients for whom it may be a trait marker) [75]. More studies are needed in a clinical setting to elucidate the effects of antidepressants with different mechanisms of action on serum zinc.

## 4. Norepinephrine (NE)

NE, also called noradrenaline (NA), is one of the principal catecholaminergic neurotransmitters that have been implicated in the monoamine hypothesis of depression and antidepressant action [3].

NE is synthesized by both the CNS and the sympathetic nervous system. In the brain, NE is produced in nuclei, of which the most important is the locus coeruleus (LC), the most extensively projecting nucleus in the brain [76, 77]. The NE projections from the LC reach brain regions such as the cortex, the hippocampus and the amygdala, which govern memory, cognition and mood [78]. Exposure to stress, which is considered to be a precipitant of depression [79], activates the LC through efferents from the corticotropin-releasing factor (CRF) system [80]. Therefore, LC projections and inputs have received great attention with regard to depressive disorders.

NE is synthesized from the precursor amino acid tyrosine by a series of enzymatic steps. Tyrosine is transported to the CNS from the blood by means of an active transport pump. First, tyrosine is converted into DOPA by tyrosine hydroxylase, the rate-limiting enzyme in NE synthesis. Then, DOPA is converted into dopamine (DA) by DOPA decarboxylase. The third enzymatic step is the conversion of DA into NE by dopamine *β*-hydroxylase. While the first two steps occur predominantly in the cytoplasm, the last one takes place mainly in the synaptic vesicles. NE is degraded to inactive metabolites by either MAO [81], which is localized in the mitochondrial outer membrane [82], or catechol-O-methyltransferase (COMT) [81], which is located intracellularly [83]. The other mechanism terminating synaptic NE action is NET, which belongs to the neurotransmitter/sodium symporter family and which is localized on the presynaptic noradrenergic nerve terminals [84, 85]. Following reuptake into the presynaptic neuron by NET, NE can be either stored again in the synaptic vesicles by the VMAT2 [86] or degraded by enzymes. NE exerts its action via the family of G protein-coupled receptors (*α* and *β* subtypes). Of particular interest in terms of antidepressant pharmacology are *α*_2_ adrenergic receptors, which can act as presynaptic autoreceptors, and thereby regulate NE release [87, 88]. Many antidepressants target NET (e.g., SNRIs such as venlafaxine, duloxetine, and milnacipran; norepinephrine-dopamine reuptake inhibitors (NDRIs) such as bupropion; selective norepinephrine reuptake inhibitors (NRIs) such as reboxetine; and TCAs such as imipramine and amitriptyline) and/or adrenergic receptors (e.g., drugs with prominent *α*_2_-blocking properties, such as mirtazapine and mianserin). Moreover, chronic administration of antidepressants induces adaptive changes in the adrenergic receptors (i.e., *β*-downregulation [89, 90], *α*_1_-upregulation [91, 92], or *α*_2_-downregulation [90]).

### 4.1. Interactions between Zinc and the Noradrenergic System

Despite a high sequence identity between DAT, which possesses an endogenous, high-affinity zinc-binding site, and NET, the latter does not possess a zinc-binding site [41]. NET contains two of the three zinc coordinating residues found in DAT, but monoamine reuptake by NET is not affected by zinc. However, if the third DAT zinc coordinating residue (H193) is introduced into NET (position 189), NET becomes susceptible to inhibition by zinc [93]. It has been reported that the expression of NET was decreased in the locus coeruleus by the cooccurrence of social isolation and zinc deficiency compared with zinc deficiency alone, and that this change was accompanied by an increase in the blood concentration of 3-methoxy-4-hydroxyphenylglycol (MHPG), an NE metabolite [94]. It was long assumed that the peripheral measurement of MHPG reflects similar activity of the CNS NE system; however, further studies have shown that the brain contains about 20% of the peripheral levels of this metabolite [81].

Apart from NET, zinc targets other NE system components, including the *α*_1_ and *β*_2_ receptors, which are expressed in the CNS and which are implicated in the pathophysiology of depression and antidepressant action [95]. The ion interacts with the *α*_1A_-adrenoceptor with affinities in the low *μ*M range and acts as a negative allosteric modulator for this receptor [96]. Moreover, it acts as a positive allosteric modulator for NE, which suggests that it may bind to two distinct binding sites of the *α*_1A_ [96]. Zinc is also a positive allosteric modulator of agonist binding for the *β*_2_-adrenoreceptor [97, 98].

Zinc administration may affect levels of central NE and/or levels of its metabolites. A 3-day i.p. administration of 5 mg zinc acetate/kg body weight resulted in a significant increase in NE levels in the whole brains of rats, whereas DA levels slightly decreased [99]. Wallwork et al. [100] observed that the NE level increased in the brains of rats fed a zinc-deficient diet for 9-10 days compared with pair-fed or ad libitum-fed control rats, which received the same diet as the zinc-deficient group but were given zinc acetate via their drinking water. When measured by in vivo microdialysis, NE concentration was found to have decreased in the paraventricular nucleus (PVN) of the hypothalamus of rats fed the zinc-deficient diet for 2 weeks compared with rats fed the zinc-adequate diet [101]. Moreover, decreased concentrations of NE and its metabolite 3,4-dihydroxyphenylglycol (DHPG) were found in the PVN of rats subjected to a 2-week zinc-deficient diet, and this was associated with increased NE activity measured as a higher ratio of DHPG to NE [102]. These data indicate that dietary zinc deficiency may also affect central NE levels. Furthermore, prenatal exposure to zinc oxide nanoparticles was found to increase the level of normetanephrine, another NE metabolite, in the hippocampus of mouse offspring, as well as to decrease the MHPG level in the hypothalamus and cerebellum; however, it did not affect the NE level in any of the brain regions examined [103].

### 4.2. Interactions between Zinc and Antidepressants Targeting the Noradrenergic System

Zinc was found to be active in a number of preclinical tests (e.g., FST, TST) and models of depression [9–13, 15]. Low, ineffective doses of zinc administered together with low, ineffective doses of imipramine were active in the FST [104] and the TST [13]. However, the combined treatment of subeffective doses of zinc and reboxetine did not result in a significantly reduced immobility time in the FST [52]. Moreover, an increase in the swimming parameter but not in the climbing parameter in the FST was observed following zinc administration [52]. It is suggested that in the FST the swimming parameter is connected with serotonergic neurotransmission, and the climbing parameter is linked to noradrenergic neurotransmission [63]. Therefore, the above-mentioned results suggest that the noradrenergic system is not involved in the antidepressant-like activity of zinc. However, this may be a phenomenon observed only in the FST, as combined treatment, involving the administration of subeffective doses of zinc chloride together with subeffective doses of desipramine, a TCA with better selectivity for NE reuptake, induced a significant reduction in immobility time in the TST [13].

Zinc deficiency induces depression-like behavior [21, 22, 24, 25, 27]. Animals subjected to a zinc-deficient diet and treated with an acute injection of imipramine or reboxetine displayed increased immobility time in the FST compared with animals treated with a zinc-adequate diet and the respective antidepressant [65]. Also, increased immobility time was observed in mice fed a zinc-deficient diet after chronic reboxetine administration, compared with mice fed a zinc-adequate diet and treated with the antidepressant drug, whereas chronic imipramine treatment in mice subjected to either a zinc-deficient or a zinc-adequate diet resulted in no significant differences in immobility time between the groups [65]. These results indicate that zinc deficiency alters responsiveness to antidepressants targeting the NE system.

The zinc-sensing GPR39 receptor is involved in the pathophysiology of depression and antidepressant action [32]. Chronic treatment with reboxetine but not with imipramine induced upregulation of the GPR39 protein in the frontal cortex of mice [69]. GPR-39 knockout mice display depressive-like behavior [33]. While imipramine and reboxetine reduced immobility time in the FST in wild-type (WT) mice, they were inactive in this test in GPR39 knockout mice, suggesting that the GPR39 receptor is required for the antidepressant effect of antidepressants targeting the noradrenergic system [71]. Moreover, administration of *α*-methyl-p-tyrosine, an inhibitor of tyrosine hydroxylase, the rate-limiting enzyme in NE synthesis, which causes inhibition of NE and DA synthesis, induced upregulation of the GPR39 protein level in the frontal cortex after a 3-day administration and downregulation of this receptor in the hippocampus after a 10-day administration [55], indicating a possible role of the GPR39 receptor in NE transmission.

The first communication suggesting a beneficial effect of zinc supplementation in clinical depression showed that patients receiving TCAs (clomipramine, amitriptyline) together with zinc displayed significantly reduced depression scores compared with patients receiving TCAs and placebo [6]. Furthermore, a randomized, double-blind, placebo-controlled study showed that zinc supplementation augments the efficacy of imipramine in treatment-resistant patients [18]. Whereas zinc supplementation of therapy with SSRIs was found to be beneficial in depressed patients [7], so far no study has examined the effects of the joint administration of zinc and an antidepressant selectively blocking NE reuptake.

### 4.3. Effects on Zinc Levels of Antidepressants Targeting the Noradrenergic System

Repeated treatment with imipramine slightly increases the zinc level in the hippocampus and slightly decreases it in the cortex, the cerebellum, and the basal forebrain, but it does not affect the serum zinc level [73]. No significant differences were observed in the serum zinc level between mice that received a zinc-deficient diet and chronic imipramine or reboxetine treatment and mice that received a zinc-adequate diet and the respective drug treatment, while the level of the stress hormone, corticosterone, was increased in the serum of the zinc-deficient mice [65]. A clinical study in which zinc supplemented imipramine therapy found a significantly lower serum zinc level in depressed patients than in healthy volunteers. All groups demonstrated a gradual increase in zinc concentrations over the period of imipramine treatment with or without zinc supplementation. It is of note that treatment-resistant patients demonstrated lower concentrations of zinc than patients who were not resistant to treatment. Importantly, over the course of 12 weeks of imipramine treatment, a significant negative correlation was demonstrated between the Montgomery-Åsberg Depression Rating Scale and the serum zinc level, together with a concomitant increase in serum zinc in patients in remission [75]. So far no study has examined the effects of antidepressants that selectively block NE reuptake on serum zinc level in a clinical setting.

## 5. Dopamine (DA)

Dopamine (DA), another catecholaminergic neurotransmitter, has initially received less attention in relation to the original monoamine hypothesis of depression; however, in the 1970s its role was postulated [105].

In the brain, most DA-synthesizing neurons are located in the brainstem nuclei: the substantia nigra and the ventral tegmental area (VTA). VTA neurons project to the cortex via the mesocortical pathway, and to the nucleus accumbens, the hippocampus and the amygdala via the mesolimbic pathway. Projections from the substantia nigra to the dorsal striatum constitute the nigrostriatal pathway. The other pathway is the tuberoinfundibular pathway, which regulates prolactin secretion [106]. DA pathways are involved in various CNS functions, such as memory, learning, attention, movement, reward, and affect [106].

DA is synthesized from the amino acid tyrosine and is a precursor for NE. Tyrosine hydroxylase converts tyrosine into DOPA, whereas DOPA decarboxylase converts DOPA into DA. After synthesis, which occurs in the cytoplasm, DA is transported into the synaptic vesicles by VMAT2. DA is cleared from the extracellular space by DAT, which, like NET and SERT, belongs to the neurotransmitter/sodium symporter family and is inactivated by COMT and MAO. The prefrontal cortex possesses a few DATs, and DA is terminated in this region by NET [106, 107]. DA exerts its action via G protein-coupled D_1_-like (D_1_, D_5_) and D_2_-like (D_2_, D_3_, D_4_) receptors, which are widely expressed in the brain [107]. Pharmacological agents targeting DA system components are largely used in the treatment of psychiatric and neurological diseases (e.g., schizophrenia, bipolar disorder, depression, Parkinson's disease, attention deficit hyperactivity disorder). Bupropion, which belongs to the NDRIs, is among the antidepressants targeting DAT [108]. Unlike SSRIs, it has a very low rate of sexual dysfunction being experienced as a side effect [108]. Sertraline is among the SSRIs which inhibit DAT [109]; however, it remains controversial whether this action is clinically relevant. Nevertheless, adding bupropion to SSRI therapy is effective as an augmentation strategy [108], and adding bupropion to sertraline treatment is also effective, probably because of the combination of the weak DAT properties of both. It should be noted that drugs inhibiting NET cause an increase in the DA level in the prefrontal cortex. Additionally, chronic administration of antidepressants induces adaptive changes to DA receptors (D_1_-downregulation [110] and D_2_-upregulation [111]), and DA receptors are involved in the antidepressant-like effect of different compounds [95].

### 5.1. Zinc Interactions with the Dopaminergic System

The binding of zinc to DAT is long established. Extracellular zinc binds to DAT and restricts the transporter's movement through the conformational cycle, resulting in a decrease in substrate uptake. Two major coordinating histidine residues (H193 in the large second extracellular loop (ECL2) and H375 in the fourth extracellular loop (ECL4)) in the zinc-binding site of DAT were identified [93]. Next, it was shown that the zinc-binding site of DAT consists not only of H193 and H375 but also of E396 and D206 [112]. Extracellular zinc is a potent inhibitor of DAT in cells expressing human DAT [93] and in synaptosomes [113] with an IC50 in the low *μ*M range. Considering that after depolarization the concentration of zinc in the synaptic cleft is estimated to be between sub-*μ*M and 100 *μ*M [40], zinc action on DAT seems to be physiologically relevant. Also, zinc modulates the dopaminergic receptors. The ion was reported to allosterically inhibit the binding of subtype-specific antagonists of the D_1A_ and D_2L_ receptors [114]. The binding of the selective antagonists to the respective receptors was reduced in the presence of *μ*M zinc concentrations [114]. Moreover, it was shown that zinc inhibits the binding of the selective antagonist (spiperone analogue: [^3^H]methylspiperone) to D_2_-like receptors. Zinc inhibition of antagonist binding to the D_4_ receptor was found to be noncompetitive, whereas in the case of D_2L_ and D_3_ it was found to be a competitive allosterism [115]. Zinc was shown to modulate antagonistic binding to the entire D_2_-like subfamily with concentrations in the low *μ*M range [115]. Furthermore, it was found that zinc binding to H394 and H399 on the dopamine D_2_ receptor contributes to the allosteric regulation of antagonist binding [116].

An increased DA level in the hippocampus of mouse offspring following prenatal exposure to zinc oxide nanoparticles was observed [103]. This change was associated with increased levels of DA metabolites: homovanillic acid in the prefrontal cortex and in the hippocampus, as well as 3,4-dihydroxyphenylacetic acid (DOPAC) in the prefrontal cortex [103]. Prenatal exposure to zinc oxide nanoparticles also increased DA turnover in the prefrontal cortex, the neostriatum, the nucleus accumbens and the amygdala [103]. There were no changes in DA, DOPAC, or DOPAC-to-DA ratios in the PVN of rats subjected to administration of a zinc-deficient diet [102].

### 5.2. Interactions between Zinc and Antidepressants Targeting the Dopaminergic System

The joint administration of low, ineffective doses of bupropion and low, ineffective doses of zinc produced a significant decrease in immobility time in the TST, which suggests the involvement of dopaminergic neurotransmission in the antidepressant-like activity of zinc [13, 52]. Animals subjected to a zinc-deficient diet and treated with an acute injection of bupropion showed increased immobility time in the FST compared with animals treated with a zinc-adequate diet and the drug [65]. In addition, increased immobility time was observed in mice fed a zinc-deficient diet after chronic bupropion administration, compared with mice fed a zinc-adequate diet and given the antidepressant [65]. These behavioral changes were associated with increased serum corticosterone concentrations [65]. In animals chronically administered with bupropion, the serum zinc concentrations did not differ between those that received zinc-deficient diets and those that received zinc-adequate diets [65].

To date, no clinical study has examined the effects of the joint administration of zinc and antidepressants targeting DA reuptake or the effects of administration of antidepressants with this mechanism of action on serum zinc levels.

## 6. Link to Neural Plasticity

In 1998, after the pioneering work of Altman [117], followed by the studies of Kaplan and Hinds [118], contrary to the earlier dogma which stated that the adult nervous system does not produce new neurons, Eriksson et al. [119] demonstrated the occurrence of neurogenesis in the dentate gyrus of the hippocampus of adult humans. The discovery had a great impact on the way of thinking about the human brain and diseases. Preclinical and clinical studies have suggested that the pathophysiology of depression is associated with the inability of neuronal systems to exhibit appropriate plasticity [120]. It was shown that psychosocial stress causes atrophy of CA3 pyramidal cells in the hippocampus [121] and decreases neurogenesis in the dentate gyrus of adult animals [122]. It was postulated that these damaging effects of stress could contribute to the reduced volume of the hippocampus observed in depressed patients [123]. The initial report on the effect of antidepressants on hippocampal neurogenesis in the adult rats has contributed to the neural plasticity theory of depression and antidepressant action [124]. The antidepressants tested in the first series of studies aimed to examine their effects on the production of neurons in the adult rat brain included a MAO inhibitor: tranylcypromine, an SSRI: fluoxetine, and an NRI: reboxetine, as well as electroconvulsive seizures. It was shown that chronic antidepressant treatment (14–21 days in case of the above-mentioned drugs, 10 days in case of electroconvulsive seizures) significantly increased the number of cells positive for bromodeoxyuridine (BrdU), the thymidine analogue that labels DNA during the S-phase, in the dentate gyrus of the hippocampus [124], one of a few brain regions where production of neurons occurs throughout the lifetime [119]. In contrast, administration of fluoxetine for 1 or 5 days did not significantly affect the number of BrdU-positive cells [124], consistent with the finding that antidepressants require weeks to produce therapeutic effect. Angiogenesis is a process coupled to neurogenesis. It was shown that SSRIs increased human hippocampal progenitor cells and angiogenesis selectively in the anterior and central dentate gyrus suggesting angiogenesis as a therapeutic strategy [125]. Also newer compounds, like a multimodal agent vortioxetine, after chronic treatment were shown to increase dendritic length and the number of dendrite intersections as well as to increase cell proliferation, cell survival and stimulate maturation of immature granule cells in the subgranular zone of the dentate gyrus [126]. Moreover, vortioxetine [127] but also other generations of antidepressants like SSRIs (escitalopram) [128] or SNRIs (milnacipran) [129] were found to prevent the effects of stress on hippocampal long term potentiation (LTP), one of the phenomena underlying synaptic plasticity [130]. In the next paragraph we will discuss the role of zinc in the processes related to neural plasticity and then possible interactions between zinc and monoamine-based antidepressants in the context of neural plasticity.

### 6.1. Zinc and Neural Plasticity

Adult male rats fed with a zinc-deficient (1 mg zinc/kg) diet for 3 weeks had ca. 50% fewer stem cells positive for Ki67, a marker for proliferation (which is expressed in cells that are in all active phases of the cell cycle but not expressed in G0 phase), in the subgranular zone and granular cell layer of the dentate gyrus [131], suggesting that zinc is required for neuronal precursor cell proliferation. When cultured human Ntera-2 (NT2) neuronal precursor cells were deprived of zinc using the chelator N,N,N,N-tetrakis(2-pyridylmethyl)ethylenediamine (TPEN), a significant decrease in cellular proliferation, as measured by BrdU uptake was observed [131]. When rats were fed a zinc-deficient (2.7 mg zinc/kg) diet for 6 weeks, a decrease in the number of progenitor cells and immature neurons was observed in the dentate gyrus. The number of progenitor cells and immature neurons was restored after a 2-week reversal to a zinc-adequate (44 mg zinc/kg) diet. Moreover, a 1-week treatment with the zinc chelator, clioquinol, decreased zinc staining in the hippocampus and reduced the number of progenitor cells. Furthermore, zinc chelation reduced hypoglycemia-induced progenitor cell proliferation and neurogenesis [132]. Additionally, ZnT-3 knockout mice, which lack vesicular zinc [133], had significantly fewer proliferating progenitor cells and immature neurons after hypoglycemia [132]. Also, mice fed a zinc-deficient (0.85 mg zinc/kg) diet for 5 weeks displayed reduced vesicular zinc in CA1 and CA3 regions of the hippocampus, which was associated with a reduction in proliferating cells labeled with BrdU and immature neurons labeled with doublecortin (DCX) immunoreactivity in the dentate gyrus. The processes of DCX-positive neurons were shortened and flexuously went through into the granular cell layer in the zinc-deficient hippocampus, suggesting that zinc deficiency, in addition to stem cell proliferation, impairs neuronal differentiation [134]. These converging data provide evidence for the important role of zinc in hippocampal neurogenesis. Zinc was also found to participate in the regulation of angiogenesis, a process which accompanies neurogenesis, through effects on pro- and antiangiogenic factors [135]. In NT2 cells deprived of zinc an increase in caspase 3/7-dependent apoptosis was observed, which was associated with a nuclear translocation of the tumor suppressor protein p53, a transcription factor involved in the regulation of cell cycle and apoptosis. The examination of p53 downstream target genes in zinc-deficient NT2 cells revealed the induction of a variety of proapoptotic genes in the initial phase of zinc restriction (6 h after TPEN treatment), like reprimo gene, which induces G2 cell cycle arrest, and, in the latter phase (18 h after TPEN treatment), the induction of proapoptotic genes such as transforming growth factor-*β* (TGF-*β*) and retinoblastoma-1 (Rb-1), as well as cellular protection genes such as glutathione peroxidase (GPx), suggesting that prolonged restriction of zinc induces mechanisms for cellular protection [131]. The apoptosis proteins, including Fas, Fas ligand (FasL), apoptosis inducing factor (AIF), and caspase-3, were significantly activated in zinc-deficient mouse hippocampus [134]. These data show that zinc deficiency induces neuronal apoptosis. Studies with human induced pluripotent stem (iPS) cells differentiated into motor neurons demonstrated that expression of zinc homeostasis regulating genes, from the zinc transporters (ZnTs) family and metallothioneins (MTs), is regulated at various stages of differentiation, that is, at stages of iPS cells, embryoid bodies, neural rosettes, neuronal stem cells and motor neurons. When iPS cells were differentiated using zinc-deficient medium the number of neuronal stem cells clusters was reduced. In this study, at this stage no differences in markers for apoptosis were observed, but increase in the number of cells undergoing apoptosis was observed at the stage of embryoid bodies. Importantly, under zinc deficiency conditions electrophysiological recording revealed a reduction of glutamate, both AMPAR and NMDAR currents, and a reduction in the total number of cells responding to glutamate stimulation [136]. Taken together, the data show that zinc may have an impact not only on neurogenesis, but also on synaptogenesis. Zinc is necessary for the structural integrity of the postsynaptic density (PSD), a specialized electron dense region of the postsynaptic membrane of excitatory synapses. The ion was found to influence the recruitment of ProSAP/Shank proteins, which are observed at PSD early during synaptogenesis, to PSD during the course of synaptogenesis and synapse maturation [137]. It was shown that the overexpression of zinc-sensitive ProSAP1/Shank2 or ProSAP2/Shank3 increases synapse density, whereas depletion of synaptic zinc along with the knockdown of zinc-insensitive Shank1 causes the rapid disintegration of PSD and the loss of several postsynaptic molecules including NMDARs [137].

NMDARs and AMPARs, which are well known to mediate synaptic plasticity, are among targets for zinc released from glutamatergic vesicles. NMDAR functions as a heteromeric complex composed of four subunits surrounding a central cation-selective pore. Three major subtypes of NMDAR subunits have been identified: GluN1, GluN2A-D, and GluN3A-B [138]. The most widely expressed NMDAR is composed of two glycine binding GluN1 subunits and two glutamate-binding GluN2 subunits (GluN2B or GluN2A or a mixture of the two). Zinc inhibits NMDAR and two different mechanisms of action were described: a voltage-independent, noncompetitive (allosteric) inhibition, responsible for reducing channel-opening frequency, and voltage-dependent inhibition, representing an open channel blocking effect of zinc [36, 139]. The comparison of GluN1/GluN2A and GluN1/GluN2B receptors showed that the voltage-dependent inhibition is similar in both types of receptors but the voltage-independent zinc inhibition is subunit-specific, with an affinity ranging from low nM for GluN1/GluN2A receptors to about 1 *μ*M for GluN1/GluN2B receptors and ≥10 *μ*M for GluN1/GluN2C and GluN1/GluN2D receptors [36]. Recent study using GluN2A-H128S knockin mice, in which the high-affinity (nM) zinc inhibition of NMDAR is specifically eliminated, indicated that under resting conditions zinc levels are too low for tonic inhibition of GluN2A at hippocampal mossy fiber synapses, the most zinc enriched synapses in the brain, which is in contrast to the earlier belief that zinc levels are high enough to tonically occupy the nM zinc sites. The study showed that following neuronal activity zinc increases transiently in the synaptic cleft, where it has a short lifetime (<2 ms at Schaffer collateral-CA1 synapse; <30–40 ms at mossy fiber-CA3 synapses) and reaches concentrations sufficient to occupy the high-affinity (nM), but not the low affinity (*μ*M), zinc sites on postsynaptic receptors [140]. It should be noted, however, that the concentration of zinc reached in the synaptic cleft has long been a matter of debate and has been estimated to range from sub-*μ*M to over 100 *μ*M according to different groups [40, 140]. Moreover, it is plausible that pathological conditions associated with intense neuronal activity will affect its concentration. Zinc was also found to modulate another subtype of ionotropic glutamatergic receptors—AMPARs. These receptors are composed of four types of subunits: GluA1, GluA2, GluA3 and GluA4 [141]. GluA2-lacking-AMPAR are permeable for Ca^2+^, which has an important consequences for neural plasticity [142, 143]. At lower concentrations (≈30 *μ*M) zinc potentiates AMPAR-induced currents, but at higher (mM) concentrations it inhibits them [144].

Stimulation of NMDAR as well as stimulation of GluA2-lacking-AMPAR results in the Ca^2+^ influx and induction of intracellular signaling pathways including: Ca^2+^-calmodulin-dependent protein kinase (CaMK), cAMP response element binding protein (CREB), BDNF and its receptor, tropomyosin-related kinase B (TrkB) [145]. BDNF binding to TrkB induces receptor dimerization and triggers its intrinsic tyrosine kinase activity, which results in activation of signaling cascades that lead to enhanced neuronal survival and differentiation. BDNF signaling via the TrkB receptor divides into three pathways, all of which converge on CREB, which in turn upregulates gene expression. These pathways include Ras-microtubule-associated protein kinase (MAPK)/extracellular signal regulated kinase (ERK) pathway; phosphatidyl inositol-3 kinase (PI3K)/Akt kinase pathway; and phospholipase C- (PLC-) *γ*/CaMK or protein kinase C (PKC) pathway [145]. Exposure to *μ*M zinc concentrations was found to transactivate TrkB in cultured cortical neurons [146]. The same group, using brain sections isolated from ZnT-3 knockout mice, surprisingly found increased immunoreactivity for activated TrkB in axons but not synaptic boutons of hippocampal mossy fibers, which suggested that vesicular zinc does not activate TrkB in hippocampal mossy fiber axons under physiological conditions [147]. However, the latter study does not contradict the previous one, because under different conditions zinc concentrations may vary and therefore trigger different effects. Mice fed with a zinc-deficient diet (0.85 mg zinc/kg) for 5 weeks have decreased protein levels of calmodulin and phosphorylated CaMKII and CREB in the hippocampus, which was associated with learning and memory impairments in the Morris water maze [148]. In turn, exposure to *μ*M zinc concentrations was found to activate MAPK and ERK1/2 in rat cultured neurons [149] and an increase in ERK1/2 phosphorylation was observed after chronic treatment with zinc [150]. Also activation of GPR39 receptor was found to induce signaling pathways involved in neural plasticity [32]. GPR39 signals through G*α*_s_, G*α*_q_ and G*α*_12/13_ proteins and displays high constitutive activity via G*α*_q_ and G*α*_12/13_ but not via G*α*_s_ pathway [151]. Downstream kinases of G*α*_s_ (protein kinase A (PKA)) and G*α*_q_ (CaMK, MAPK) activates CREB, whereas G*α*_12/13_ stimulates SRE-mediated transcription [8]. Application of *μ*M zinc concentrations to hippocampal slices induced phosphorylation of ERK1/2 and CaMKII. Application of the G*α*q inhibitor reduced the zinc-dependent phosphorylation of ERK1/2 and CaMKII, suggesting that activation of G*α*q pathway is necessary for the zinc-dependent phosphorylation of these kinases in CA3 region of the hippocampus [152].

The above-mentioned data, although not exhaustive, point to the role of zinc in neuro- and synaptogenesis as well as to the relationship between zinc and signaling pathways critical for neural plasticity. Importantly, elements of these signaling pathways were found to participate in the antidepressant-like activity of zinc and/or were affected by the condition of zinc deficiency which concomitantly induced depressive-like behavior [153].

### 6.2. Zinc, Monoamine-Based Antidepressants, and Neural Plasticity: Possible Interactions

**Figure 1:**
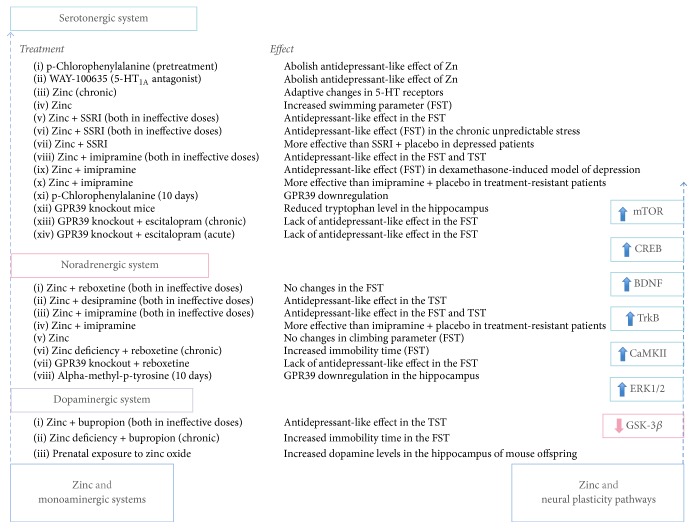
The summary of the findings related to interactions between zinc and monoaminergic systems and neural plasticity pathways.

As it was shown in the STAR^*∗*^D study the average time required to achieve remission is 6-7 weeks [4]. A delayed onset of action of conventional antidepressants and time needed to achieve response/remission are among the main reasons underlying the need for novel antidepressant treatments. Evidence indicates that neural adaptations are involved in the mechanism of action of conventional antidepressants after chronic administration [154]. In contrast, a single infusion of subanaesthetic doses of the NMDAR antagonist ketamine exerts rapid and sustained antidepressant effects in patients with treatment-resistant depression [155]. Ketamine is NMDAR channel blocker, which enters the open channel in an activity-dependent manner and binds with low *μ*M affinity. After channel closure, ketamine can become blocked within the pore, therefore, belongs to “trapping blockers”, whose block is slow to reverse [156]. As we have already mentioned, zinc, depending on its concentration, can bind to the subunits of the NMDAR or block the channel. Allosteric inhibition of NMDAR by zinc was found to be highly subunit-specific with an affinity ranging from low nM for GluN1/GluN2A receptors to about 1 *μ*M for GluN1/GluN2B receptors and ≥10 *μ*M for GluN1/GluN2C and GluN1/GluN2D receptors [36]. NMDAR open channel block by zinc was described when the concentrations of the ion were between 20 and 100 *μ*M [156]. It was found that a low dose of ketamine, which produces antidepressant-like effect in behavioral paradigms [157], rapidly and transiently activated the mammalian target of rapamycin (mTOR) signaling pathway, leading to enhanced and sustained elevation of synaptic proteins expression (e.g., postsynaptic density protein 95 (PSD95), synapsin I or GluA1) and an increased number and function of new synapses in the prefrontal cortex of rats [158]. In contrast, antidepressants such as imipramine or fluoxetine or electroconvulsive seizures did not significantly influence mTOR signaling [158]. Recent study demonstrated that a single dose of zinc (5 mg/kg) administered 30 minutes prior to the FST produced antidepressant-like effect, which lasted up to 3 h [159]. Unlike ketamine, zinc did not produce a sustained antidepressant-like effect [159]; however, in contrast to conventional antidepressants (imipramine, fluoxetine) or electroconvulsive seizures [158], it induced a transient (observed 30 min and 3 h after the treatment) increase in the protein levels of phosphorylated mTOR and ribosomal protein S6 kinase (p70S6K). An elevated level of GluA1 and synapsin I was still observed 24 h after the zinc treatment. In addition, antidepressant-like effect of zinc in the FST was blocked by pretreatment with rapamycin, mTOR inhibitor [159], which suggest that mTOR is involved in the antidepressant-like action of zinc. Also, blockade of mTOR signaling blocked ketamine behavioral effects and induction of synaptogenesis [158]. Although further studies are needed, it is plausible that the beneficial effects of zinc as an augmentation strategy in conjunction with imipramine [18] or fluoxetine [7], that have been observed in clinical trials, result from adding zinc effects on mTOR to the effects of the above-mentioned drugs, which lack these action (Figure 1). Moreover, the activation of glycogen synthase kinase-3 (GSK-3) leads to inhibition of mTOR pathway [160]. Zinc was found to inhibit GSK-3 [161]. Zinc administered in combination with AR-A014418, GSK-3*β* inhibitor, produced synergistic effects in the FST [162], suggesting that the antidepressant-like effect of zinc depends on GSK-3, which further supports the link between zinc and mTOR with regard to depressive disorders.

Fast-acting behavioral antidepressant-like effect of ketamine depends also on the rapid synthesis of BDNF. Enhanced synthesis of BDNF was observed 30 min [163] or 1 h [164] after ketamine administration. In contrast, conventional antidepressants require weeks to induce an increase in BDNF protein expression, for example, 2 weeks of treatment with fluoxetine produced region-specific increase in BDNF mRNA, whereas BDNF protein level remained unaltered until 3 weeks of the treatment and reached significance after 3 weeks in CA1 and CA3 but not in other subregions of the hippocampus [165]. There were no changes in the level of BDNF protein in the prefrontal cortex 30 min after zinc treatment [159]. Also, 1 h after zinc administration Manosso et al. did not observe changes in BDNF protein levels in either the prefrontal cortex or hippocampus [162]. However, an increase in the level of BDNF protein in the prefrontal cortex was found 3 h after zinc treatment [159]. Ranjbar et al. [72] observed in patients with major depression that zinc supplementation of the therapy with SSRIs (fluoxetine, citalopram) reduced depressive symptoms more effectively that a respective SSRI and placebo, however, these effects were not associated with alterations in serum BDNF level. In contrast, in the study of Solati et al. [20], who examined zinc treatment as a monotherapy in overweight or obese subjects with depressive symptoms, a significant inverse correlation was observed between serum BDNF levels and depression severity. Is should be stressed that Solati et al. [20] found zinc monotherapy to be effective in reducing depressive symptoms in those subjects. In addition, dietary zinc deficiency [27] and GPR39-knockout induced decreased BDNF protein expression in brain regions (hippocampus, prefrontal cortex) [33], while administration of a GPR39 agonist (TC G-1008), in parallel to antidepressant-like effect, induced upregulation of BDNF protein in the hippocampus [34]. Thus, mTOR and BDNF pathway may be a link between zinc, depression and neural plasticity.

The proposed sequence of events triggered by ketamine in depression involves blocking of NMDAR on gamma-aminobutyric acid- (GABA-) ergic interneurons in the prefrontal cortex, which causes disinhibition and increases glutamate release, which primarily excites AMPAR, leading to activation of BDNF [145]. Ketamine was reported to increase extracellular glutamate in the prefrontal cortex as measured by the in vivo microdialysis [166]. Perfusion of the CA1 regions of the hippocampus by zinc was found to decrease glutamate concentration in the perfusate [167]. Extracellular glutamate concentration was increased in the hippocampus of the zinc-deficient rats [168]. Also our study showed an increase in evoked glutamate release in the prefrontal cortex of the zinc-deficient rats [169]. These studies suggest that zinc, unlike ketamine, inhibits glutamate release.

Three rapid acting antidepressant agents (ketamine, metabotropic glutamate mGlu_2/3_ receptor antagonist LY341495, and NMDAR glycine site agent GLYX-13) were found to rapidly increase the levels of the phosphorylated (activated) forms of ERK and BDNF release in rat primary cortical culture neurons [170], showing that ERK signaling is an important step in antidepressant action. An increase in ERK1/2 phosphorylation was observed after chronic (30 days) treatment with zinc (administered via drinking water that contained 300 mg of zinc chloride/L) and was associated with antidepressant-like effect in the FST. Moreover, it was accompanied by an increase in total glutathione levels in the hippocampus and cerebral cortex [150].

Of note, relationships between altered zinc homeostasis, increased oxidative/inflammatory status, and NMDAR function were implicated in depressive disorders [47, 171]. It was shown that proinflammatory cytokines, such as IL-1*β*, and reactive oxygen species can enhance the activity of IDO, which catabolizes tryptophan into kynurenine, which is further catabolized into kynurenic acid and quinolinic acid [46]. Because kynurenic acid is an endogenous antagonist, whereas quinolinic acid is a strong agonist of the NMDAR, IDO activation can lead to abnormal function of the NMDAR. Dietary zinc deficiency-induced depression-like behavior with concomitant upregulation of the NMDAR [27] and oxidative as well as inflammatory parameters (IL-1*α*, IL-1*β*) were generally enhanced in the tissue (serum, prefrontal cortex, and hippocampus) of the zinc deprived rats [169]. Also the study of GPR39 knockout mice, which display depressive-like behavior, showed immune malfunction: reduced thymus weight, reduced cell viability of splenocytes and reduced proliferative response of splenocytes [172]. Further studies are needed to elucidate changes within the immune system of GPR39 knockout mice as well as effects of antidepressants on zinc deficiency-induced and GPR39-knockout-induced immune alterations, that may be linked to neural plasticity events.

It was shown that 5-HT_1A_ knockout mice were insensitive to the effects of chronic fluoxetine administration in the novelty suppressed feeding test (NSF), which demonstrates changes in behavior in a response to chronic, but not acute antidepressant treatment, but were responsive to imipramine and desipramine. Moreover, when wild-type and 5-HT knockout mice were injected with BrdU, after a 27-day treatment with fluoxetine, imipramine or vehicle, fluoxetine caused a doubling of BrdU-labeled cells in the dentate gyrus of the hippocampus in wild-type mice but had no effect in 5-HT knockout mice. These results indicate that 5-HT_1A_ receptors are required for fluoxetine-induced neurogenesis [173]. As it was discussed earlier, 5-HT_1A_ receptors are involved in the antidepressant-like activity of zinc. This observation may provide another route linking zinc and depression with regard to neural plasticity.

### 6.3. Future Perspectives

As it has been discussed, zinc is able to enhance the effects of conventional, that is, monoamine-based, antidepressants, not only in preclinical paradigms, but also in clinical setting. Studies conducted so far point to the positive effects of zinc supplementation to the therapy with SSRIs or TCAs. More clinical studies are needed that will elucidate the possibility of augmentation with zinc a therapy involving antidepressants with different mechanisms of action (e.g., SNRIs, NRIs, or NDRIs). Given the involvement of zinc in processes related to neural plasticity, it is plausible that the beneficial effects of zinc in depressed patients result from activation of signaling pathways associated with neural plasticity events.
